# Histopathological Features of Aspirated Thrombi after Primary Percutaneous Coronary Intervention in Patients with ST-Elevation Myocardial Infarction

**DOI:** 10.1371/journal.pone.0005817

**Published:** 2009-06-05

**Authors:** Miranda C. Kramer, Allard C. van der Wal, Karel T. Koch, Saskia Z. Rittersma, Xiaofei Li, Hanneke P. Ploegmakers, José P. Henriques, René J. van der Schaaf, Jan Baan, Marije M. Vis, Martin G. Meesterman, Jan J. Piek, Jan G. Tijssen, Robbert J. de Winter

**Affiliations:** 1 Department of Cardiology, Academic Medical Center, University of Amsterdam, Amsterdam, the Netherlands; 2 Department of Pathology, Academic Medical Center, University of Amsterdam, Amsterdam, the Netherlands; Uppsala University, Sweden

## Abstract

**Background:**

Plaque disruption with superimposed thrombus is the predominant mechanism responsible for the onset of acute coronary syndromes. Studies have shown that plaque disruption and thrombotic occlusion are frequently separated in time. We established the histopathological characteristics of material aspirated during primary percutaneous coronary intervention (PCI) in a large consecutive ST-elevation myocardial infarction (STEMI) population.

**Methodology/Principal Findings:**

Thrombus aspiration during primary PCI was performed in 1,362 STEMI patients. Thrombus age was classified as fresh (<1 day), lytic (1–5 days), or organized (>5 day). Further, the presence of plaque was documented. The histopathological findings were related to the clinical, angiographic, and procedural characteristics. Material could be aspirated in 1,009 patients (74%). Components of plaque were found in 395 of these patients (39%). Fresh thrombus was found in 577 of 959 patients (60%) compared to 382 patients (40%) with lytic or organized thrombi. Distal embolization was present in 21% of patients with lytic thrombus compared to 12% and 15% of patients with fresh or organized thrombus.

**Conclusions/Significance:**

Material could be obtained in 74% of STEMI patients treated with thrombus aspiration during primary PCI. In 40% of patients thrombus age is older than 24 h, indicating that plaque disruption and thrombus formation occur significantly earlier than the onset of symptoms in many patients.

## Introduction

Acute ST-elevation myocardial infarction (STEMI) is caused by occlusion of a coronary artery as a result of coronary atherosclerotic plaque disruption with superimposed luminal thrombus.[1] Many plaque disruptions are initially covered by mural thrombi without causing clinical symptoms. These mural thrombi may organize over time, a process characterized by ingrowth of smooth muscle cells and overgrowth of endothelial cells. Organized mural thrombi may entirely be incorporated in the atherosclerotic lesion, whereby the integrity of the vessel wall is restored. These so called healed plaque ruptures are found very frequently in coronary arteries at autopsy.[2]–[4] In other patients, atherosclerotic plaque disruption with mural thrombosis leads to a process of repeated or ongoing thrombosis, which ultimately results in an acute coronary syndrome.

We recently described the composition and age of aspirated thrombi in a small cohort of STEMI patients treated with primary percutaneous coronary intervention within 6 h of onset of symptoms. We demonstrated that in approximately 50% of these STEMI patients, coronary thrombi were days or even weeks old.[5] The aim of the present study is to establish the histopathological characteristics and age of material aspirated during primary PCI in a much larger consecutive STEMI population, to identify predictors of successful thrombus aspiration and thrombus age, and to confirm the concept that there is a heterogeneous time course of different processes leading to the occlusive thrombotic event.

## Materials and Methods

### Setting

Our institution is a large referral hospital with an annual PCI volume of 2,400 procedures, 600 of which are primary PCIs. Patients are eligible for primary PCI when they have symptoms of an acute STEMI accompanied by an electrocardiogram with ST-segment elevation of ≥0.2 mV in two or more contiguous leads and present within 12 hours after the onset of symptoms. PCI is performed by standard techniques through the femoral or radial route. Since August 2001, thrombus aspiration has been routinely performed when technical feasible as determined by the operator. Three different systems have been used: the 7F Rescue catheter (Boston Scientific/Scimed, Inc, Maple Grove, Minn), the 6F Export aspiration catheter (Medtronic Vascular Incorporation, Santa Rosa, CA), and the 6/7F Proxis™ embolic protection device (St. Jude Medical, St. Paul, MN). The choice of the aspiration device is at the discretion of the operator. Pharmacologic treatment before PCI includes the administration of aspirin in a loading dose of 300 mg, unfractionated heparin 5,000–10,000 IU, and clopidogrel in a loading dose of 300 or 600 mg. The use of glycoprotein IIb/IIIa inhibitors and anti-thrombotic medications is at the discretion of the operator. Clinical, angiographic, and procedural characteristics of all PCI-procedures are prospectively collected in an electronic database.

### Study design

The study cohort consists of all patients in whom thrombus aspiration was performed in adjunct to the conventional primary PCI between August 2001 and January 2008.

Information about baseline characteristics (gender, patient age, risk factors, cardiac history, medication, etc.), procedural characteristics, angiographic characteristics (lesion length, bifurcation, chronic total occlusion, stent implantation, procedural success), and the use of thrombus aspiration devices (type of device, device passage, angiographic result, material obtained) was obtained from the electronic database. Angiographic characteristics such as distal embolization, pre- and post-procedural TIMI flow, lesion length, and residual stenosis have been prospectively recorded by the operator by visual assessment immediately after the procedure. In this study informed patient consent was not acquired, because thrombus aspiration and the histopathological assessment of aspirated material were part of routine clinical practice. In addition, because of the study being part of routine clinical practice nor a formal waiver under the description of Record-based Research from or approval by the local Medical Ethical Committee were required for this study.

Aspirated material was fixed in formalin immediately after thrombus aspiration and was sent to the department of cardiovascular pathology. Material was fixed for at least 24 hours in 10% neutral buffered formalin and embedded in paraffin. The paraffin embedded material was serially sectioned, cut, and mounted on glass slides at ≥6 levels. The sections were stained with Hematoxylin and Eosin (H&E) for light microscopy. Histopathological analyses were performed by an experienced cardiovascular pathologist (A vd W) while blinded to the clinical characteristics of the patients, angiographic findings, and the result of the PCI-procedure. The sections were analysed for the presence or absence of aspirated material. If sufficient material was available (≥1 mm^2^) and thrombus was present, thrombus age was classified into 3 groups according to previously published and histopathologically accepted definitions.[3], [5], [6] Fresh thrombus (less than 1 day) was completely composed of layered patterns of platelets, fibrin, erythrocytes and intact granulocytes. Lytic thrombus (between 1 and 5 days) shows areas of colliquation necrosis and karyorrhexis of granulocytes. Organization of thrombus (more than 5 days) is characterized by areas of ingrowth of smooth muscle cells, with or without depositions of young connective tissue and ingrowth of capillary vessels. Thrombus material with a heterogeneous composition was graded according to the age of the oldest components. Plaque was identified on the basis of either “soft plaque” material (extracellulair lipids, macrophage foam cells, and cholesterol crystals), calcific deposits, fibrous or fibro-elastic tissue, or combinations of these components.

### Statistical analysis

Continuous data were expressed as mean±standard deviation and categorical data as frequencies, unless otherwise noted. Differences between patient groups were tested with Student's *T* test or the Mann-Whitney U statistic test, as appropriate. Categorical variables were compared with the use of Fisher's Exact test. Multivariate logistic regression models were performed to identify independent predictors of the presence of aspirated material and of thrombus age. Variables were entered if the p value was less than .10 and removed if the p value was more than .10. All tests were two-sided, and p values of less than 0.05 were considered statistically significant. Statistical analysis was performed with the Statistical Package for Social Sciences software (SPSS 12.0 for Windows, SPSS Inc, Chicago, IL).

## Results

### Study cohort

**Figure 1:**
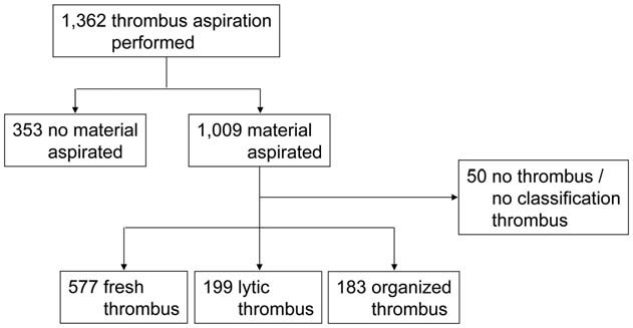
Study flow chart.

Mechanical thrombus aspiration during primary PCI was performed in 1,362 patients. Histopathologically confirmed material was obtained in 1,009 patients (74%). In the remaining 353 patients (26%) material could not be identified (Figure 1).

### Succesfully thrombus aspiration

Clinical, angiographic and procedural characteristics of the patients with and without histopathologically confirmed material are summarized in Table 1. Patients without aspirated material were older compared to patients with aspirated material. Further, patients without aspirated material more often had bifurcated and calcified lesions (13.3% versus 9.0% and 19.3% versus 14.2%). The presence of post-procedural TIMI 3 flow was similar in patients without and patients with aspirated material (91.1% versus 90.1%, p = 0.67). Distal embolization occurred significantly less often in patients in whom no material was aspirated (7.7% versus 14.7%, p = 0.002).

**Table 1:** Clinical, angiographic and procedural characteristics of the 1,362 patients treated with thrombus aspiration during primary PCI.

	Material obtained	No material obtained	P value
	(n = 1,009)	(n = 353)	
Male	748 (74)	247 (70)	0.14
Age (years)	59±13	62±12	0.001
Diabetes mellitus	100 (10)	34 (10)	0.92
Hypercholesterolaemia	205 (20)	76 (22)	0.65
Current smoking	496 (49)	163 (46)	0.35
Hypertension	313 (31)	111 (31)	0.89
Previous MI	90 (9)	33 (9)	0.83
Previous CABG)	17 (2)	4 (1)	0.62
Previous PCI	64 (6)	21 (6)	0.90
Shock	118 (12)	37 (11)	0.56
Total ischemic time (h)†	3.7±3.4	3.8±3.2	0.32
Infarct related artery LAD	436 (43)	143 (41)	0.38
Pre-procedural TIMI flow 0–1^§^	846 (85)	290 (84)	0.49
Multivessel disease^§^	283 (27)	96 (28)	0.78
Lesion type class C‡^§^	353 (37)	127 (40)	0.42
Bifurcation^§^	91 (9)	46 (13)	0.04
Ostial^§^	74 (7)	20 (6)	0.33
Calcified^§^	143 (14)	68 (19)	0.03
Lesion length (mm)^§^	16.6±6.2	15.4±5.8	<0.0001
Stent placement	937 (93)	326 (92)	0.72
Stent length (mm)	19.3±5.3	18.3±4.8	0.03
Stent diameter (mm)	3.6±0.5	3.4±0.5	<0.0001
Post-procedural TIMI flow 3^§^	898 (90)	314 (91)	0.67
Residual stenosis ≥20%^§^	35 (4)	14 (4)	0.62
Distal embolization^§^	147 (15)	27 (7)	0.001

Continous variables are presented as means and standard deviations and categorical data are presented as frequencies (counts) and percentages.

LAD = left anterior descending coronary artery; TIMI = Thrombolysis In Myocardial Infarction; § = By visual assessment by the operator; † = Time between onset of symptoms and needle time; ‡ = According to the ACC-AHA classification.

Univariate analysis showed a significant association between the presence of aspirated material and patient age, bifurcated lesions, and calcified lesions. Multivariate analysis identified patient age (0.65 [95% CI 0.51 to 0.83], p = 0.001) and occlusion of a bifurcation lesion (0.65 [95% CI 0.44 to 0.95], p = 0.024) as independent predictors of the presence of aspirated material (data not shown).

### Thrombus age

Thrombus could not be identified or thrombus could not be classified according to age, in 50 of the 1,009 patients with aspirated material. These patients were excluded from the analysis (Figure 1). We identified a completely fresh thrombus in 577 of the 959 patients (60%). Lytic thrombus was found in 199 patients (21%), 131 with only lytic thrombus and 68 with fresh and lytic thrombus. Organized thrombus was found in 183 patients (19%), 68 with only organized thrombus, 44 with fresh and organized thrombus, 40 with lytic and organized thrombus, and 31 with fresh, lytic and organized thrombus (Table 2). In total, in 382 of 959 patients (40%) aspirated thrombi showed lytic or organized changes signifying thrombus formation occurring significantly earlier than the time of onset of symptoms.

**Table 2:** Classification of thrombus age of aspirated material in 959 patients with classifiable thrombus material.

Thrombus age	Patients	Subcategory thrombus age	Patients
	(n = 959)		(n = 959)
Fresh thrombus	577 (60)		
Lytic thrombus	199 (21)	Complete lytic thrombus	131 (66)
		Fresh and lytic thrombus	68 (34)
Organized thrombus	183 (19)	Complete organized thrombus	68 (37)
		Fresh and organized thrombus	44 (24)
		Lytic and organized thrombus	40 (22)
		Fresh, lytic and organized thrombus	31 (17)

The categorical data are presented as frequencies (counts) and percentages.

Clinical, procedural, and angiographic characteristics of the patients with fresh, lytic or organized thrombus are summarized in Table 3. Total ischemic time (time between the onset of symptoms and needle time) was significantly shorter in patients with fresh thrombus (2.4 hrs versus 4.3 and 4.4 hrs, p = 0.007). There were no differences in door-to-needle time. Distal embolization occurred in 12.1% of patients with fresh thrombus, in 21.2% of patients with lytic thrombus, and in 15.2% of patients with organized thrombus (p = 0.007).

**Table 3:** Clinical, angiographic and procedural characteristics of the 959 patients with classifiable material.

	Fresh thrombus	Lytic thrombus	Organized thrombus	P value
	(n = 577)	(n = 198)	(n = 183)	
Male	439 (76)	142 (72)	132 (72)	0.32
Age (years)	59±13	61±13	58±14	0.07
Diabetes mellitus	58 (10)	20 (10)	14 (8)	0.60
Hypercholesterolaemia	117 (20)	43 (22)	35 (19)	0.81
Current smoking	288 (50)	98 (50)	93 (51)	0.98
Hypertension	192 (33)	60 (30)	50 (27)	0.28
Previous MI	43 (8)	23 (12)	15 (8)	0.19
Previous CABG	9 (2)	6 (3)	0 (0)	0.06
Previous PCI	39 (7)	13 (7)	10 (5)	0.82
Shock	64 (11)	23 (12)	27 (15)	0.42
Total ischemic time (h)†	3.3±2.4	4.0±4.3	4.2±4.4	0.007
Infarct related artery LAD	252 (44)	82 (41)	77 (42)	0.82
Pre-procedural TIMI flow 0–1^§^	488 (86)	166 (86)	153 (85)	0.99
Multivessel disease^§^	167 (29)	46 (23)	52 (28)	0.29
Lesion type class C‡^§^	205 (37)	69 (37)	60 (35)	0.90
Bifurcation^§^	54 (9)	16 (8)	16 (9)	0.85
Ostial§	39 (7)	16 (8)	18 (10)	0.39
Calcified^§^	89 (15)	28 (14)	18 (10)	0.21
Lesion length (mm)^§^	16.5±6.5	17.0±5.6	16.6±6.1	0.61
Stent placement	539 (93)	177 (89)	175 (95)	0.07
Stent length (mm)	19.1±4.8	19.5±5.8	19.7±5.8	0.98
Stent diameter (mm)	3.6±0.5	3.6±0.5	3.5±0.5	0.10
Post-procedural TIMI flow 3^§^	524 (92)	173 (88)	160 (88)	0.12
Residual stenosis ≥20%^§^	18 (3)	11 (6)	4 (2)	0.14
Distal embolization^§^	70 (12)	42 (21)	28 (15)	0.007

Continous variables are presented as means and standard deviations and categorical data are presented as frequencies (counts) and percentages.

LAD = left anterior descending coronary artery; TIMI = Thrombolysis In Myocardial Infarction; § = By visual assessment by the operator; † = Time between onset of symptoms and needle time; ‡ = According to the ACC-AHA classification.

Univariate analysis showed a significant association between the age of aspirated thrombus and total ischemic time. Multivariate analysis identified a history of hypertension (1.31 [95% CI 0.98 to 1.75], p = 0.072) and a longer total ischemic time (1.08 [95% CI 1.03 to 1.13], p = 0.001) as independent predictors for the presence of older thrombus (lytic or organized) (data not shown).

### Plaque components

Components of plaque were found in 396 of the 1,009 patients (39%) with aspirated material. In 31 patients (3%) aspirated material consists of only plaque components. Both thrombus and plaque components could be identified in 365 patients (36%). In the majority of the patients components of soft plaque were found (339/395, 86%). Histological evidence of calcific deposits and fibrous or fibro-elastic tissue was found in 105 (27%) and 104 patients (26%), respectively.

## Discussion

In this study we report the histopathological characteristics of material obtained from a large consecutive cohort of STEMI patients treated with thrombus aspiration in adjunct to conventional primary PCI. Aspirated material could histopathologically be confirmed in 74% of the patients. The obtained thrombus material showed lytic or organized changes in 40% of the patients, indicating the thrombus is older than 24 hours in a significant proportion of STEMI patients with onset of symptoms less than 12 hours before.

We found older thrombus (more than one day) in a substantial proportion of the STEMI patients (40%) and the composition of the these thrombi was often heterogeneous, showing in part features of fresh, of lytic, and organized thrombus. These results support the concept of coronary artery disease as a dynamic process. Disruption of atherosclerotic plaques may act as a stimulus for repeated or ongoing thrombosis, which ultimately progresses over a period of days or even weeks to thrombotic occlusion with a secondary fresh thrombus. Systematic histopathological analyses of the atherothrombotic material obtained with thrombus aspiration during primary PCI in large patient cohorts are limited. Our findings are in line with our previous results that describe histopathological analyses of aspirated atherothrombotic material in a smaller group of 211 STEMI patients in whom we identified older thrombus in approximately 50% of patients.[5] The insights in the mechanisms of coronary thrombosis mainly come from detailed analyses of underlying plaque morphologies in necropsy specimen from sudden death victims. Autopsy studies on the histopathology of the progression of coronary plaques demonstrated the occurrence of clinically silent coronary non-occlusive atherothrombotic events before the occlusive atherothrombotic event.[2], [7] Furthermore, multiple subclinical episodes of plaque disruption, followed by healing, are an important mechanism of atherosclerotic plaque growth.[2], [4] A close relationship has been demonstrated between the number of previous plaque disruptions and the mean percentages stenosis in the coronary artery.[2] Further, Henriques de Gouveia et al. demonstrated that histopathological evidence of plaque instability for some time was present in the majority of sudden coronary death victims. They concluded that plaque disruption with superimposed thrombus formation starts days or even weeks before the time of death.[3] Subclinical episodes of plaque disruption may have the potential to precondition the myocardium. An alternative explanation is that several subclinical plaque disruptions may results in the development of collateral vessels. Therefore, patients with successive thrombotic occlusion after several subclinical plaque disruptions may experience less acute cardiac symptoms compared with patients in whom plaque disruption immediately leads to a superimposed luminal thrombus with total occlusion. This may explain why patients with lytic or organized thrombus had a significantly longer total ischemic time compared with patients with fresh thrombus (4.3 and 4.4 hrs versus 2.4 hrs, p = 0.007). Interestingly, distal embolization was significantly different between patients with fresh, lytic or organized thrombus, with the highest percentage in patients with thrombus with lytic changes. Thrombus embolization may be less frequent when the thrombus is fresh and fragile or when the thrombus is organized and more solid and attached to the vessel wall. This pathological finding is compatible with the report by Kondo et al, who found an association between the no-reflow phenomenon and a longer ischemic time.[8] These findings suggest the presence of a time window of several days after plaque disruption when distal embolization of thrombus is most likely to occur in association with mechanical reperfusion.

In our study we aspirated atherothrombotic material in almost three-quarter of the patients in whom an aspiration device was used in adjunct to the conventional primary PCI. This is similar to the 73% rate of histopatholgically confirmed material in the EMERALD trial, the 73% rate in the TAPAS trial, and the 77% rate with the use of the Proxis™ embolic protection device.[9]–[11] Several explanations have been proposed as to why atherothrombotic material could not be aspirated in all STEMI patients. The aspiration devices may not have been efficient enough in aspirating the atherothrombotic material. Alternatively, in some patients with STEMI coronary occlusion may be caused by a non-thrombotic lesion or a subtotal atherosclerotic plaque with minimal thrombus. Consequently, in patients with lesions with little or no thrombus and no aspirated material, distal embolization was less frequent as in patients in whom material was aspirated (14.6% versus 7.7%, p = 0.001).

### Study Limitations

Except for the TAPAS study, previous studies with thrombus aspiration have been neutral or negative. Therefore, the use of a thrombus aspiration device was non-standard clinical practice during the study period and the decision to perform thrombus aspiration was at the discretion of the operator. In some patients, thrombus aspiration was not possible depending on coronary anatomy, and in 353 patients no material was obtained. However, we believe that there was no systemic selection of patients that could influence our conclusions. Secondly, we cannot exclude the possibility that atherothrombotic material was selectively aspirated from the total of atherothrombotic material in situ. In our view, this cannot be the sole explanation for the observed histopathological features of the aspirated thrombus. Thirdly, additional immunostaining to optimize visualization of smooth muscle cells in specimen of lytic or organized thrombus was not performed. The major distinction in our paper, though, is fresh versus lytic or organized thrombus, which is not affected by this limitation. Finally, full information regarding anginal symptoms in the days or weeks before the acute myocardial infarction was not available. However, our previous thrombectomy study showed no clear relation between thrombus age and the presence or absence of preinfarction angina.

### Conclusions

Our study shows that histopathologically confirmed material could be obtained in almost three-quarter of a large consecutive cohort of STEMI patients treated with thrombus aspiration during primary PCI. Forty percent of the aspirated material showed features of thrombus formation that occurred several days or even weeks earlier, which confirms that there is an unpredictable time span between onset of plaque disruption, thrombus formation, and the onset of clinical symptoms of acute myocardial infarction. In addition, our data suggest that there is a time window of several days after plaque disruption in which thrombus embolization is most likely. Knowledge of the composition and age of the thrombus, perhaps with non-invasive imaging, might be helpful in the future for the planning and choice of adjuvant therapy during primary PCI.

## References

[1] Van Der Wal AC, Becker AE (1999). Atherosclerotic plaque rupture—pathologic basis of plaque stability and instability.. Cardiovasc Res.

[2] Mann J, Davies MJ (1999). Mechanisms of progression in native coronary artery disease: role of healed plaque disruption.. Heart.

[3] Henriques de GR, Van Der Wal AC, van der Loos CM, Becker AE (2002). Sudden unexpected death in young adults. Discrepancies between initiation of acute plaque complications and the onset of acute coronary death.. Eur Heart J.

[4] Burke AP, Kolodgie FD, Farb A, Weber DK, Malcom GT (2001). Healed plaque ruptures and sudden coronary death: evidence that subclinical rupture has a role in plaque progression.. Circulation.

[5] Rittersma SZ, Van Der Wal AC, Koch KT, Piek JJ, Henriques JP (2005). Plaque instability frequently occurs days or weeks before occlusive coronary thrombosis: a pathological thrombectomy study in primary percutaneous coronary intervention.. Circulation.

[6] Murakami T, Mizuno S, Takahashi Y, Ohsato K, Moriuchi I (1998). Intracoronary aspiration thrombectomy for acute myocardial infarction.. Am J Cardiol.

[7] Arbustini E, Grasso M, Diegoli M, Morbini P, Aguzzi A (1993). Coronary thrombosis in non-cardiac death.. Coron Artery Dis.

[8] Kondo M, Nakano A, Saito D, Shimono Y (1998). Assessment of “microvascular no-reflow phenomenon” using technetium-99m macroaggregated albumin scintigraphy in patients with acute myocardial infarction.. J Am Coll Cardiol.

[9] Koch KT, Haeck JD, Van Der Schaaf RJ, Alidjan FM, Henriques JP (2007). Proximal Embolic Protection With Aspiration in Percutaneous Corononary Intervention Using the Proxistrade mark Device.. Rev Cardiovasc Med.

[10] Stone GW, Webb J, Cox DA, Brodie BR, Qureshi M (2005). Distal microcirculatory protection during percutaneous coronary intervention in acute ST-segment elevation myocardial infarction: a randomized controlled trial.. JAMA.

[11] Svilaas T, Vlaar PJ, van dH I, Diercks GF, de Smet BJ (2008). Thrombus aspiration during primary percutaneous coronary intervention.. N Engl J Med.

